# Nur77 attenuates inflammatory responses and oxidative stress by inhibiting phosphorylated IκB-α in Parkinson’s disease cell model

**DOI:** 10.18632/aging.103128

**Published:** 2020-05-13

**Authors:** Junqiang Yan, Jiarui Huang, Jiannan Wu, Hua Fan, Anran Liu, Liang Qiao, Mengmeng Shen, Xiaoyi Lai

**Affiliations:** 1 Neurological Diseases Institute, The First Affiliated Hospital, College of Clinical Medicine of Henan University of Science and Technology, Luoyang 471003, P.R. China; 2 Department of Neurology, The First Affiliated Hospital, College of Clinical Medicine of Henan University of Science and Technology, Luoyang 471003, P.R. China; 3 Department of Pharmacy, The First Affiliated Hospital, College of Clinical Medicine of Henan University of Science and Technology, Luoyang 471003, P.R. China

**Keywords:** Nur77, Parkinson’s diseases, phosphorylated IκB-α, neuroinflammation, anti-oxidant stress

## Abstract

Neuroinflammation and oxidative stress play key roles in the pathological development of Parkinson’s disease (PD). Nerve growth factor-induced gene B (Nur77) is closely related to dopamine neurotransmission, and its pathogenesis is unclear. This study aims to investigate the role and mechanism of Nur77 in a cell model of Parkinson’s disease. Silencing Nur77 with siRNA can aggravate intracellular LDH release, increase the expression of pro-inflammatory genes (such as tumor necrosis factor α, nuclear factor κB (p65), monocyte chemotactic protein 1, interleukin-6), and decrease cell survival, decrease expression of nuclear factor E2-related factor(Nrf2), heme oxygenase 1, NADPH quinineoxidoreductase-1. Cytosporone B (Nur77 agonist) has the opposite effect to Nur77 silencing. PDTC (NF-κB inhibitor / antioxidant) can also inhibit pro-inflammatory genes to a similar degree as Cytosporone B. Phosphorylated IκB-α can be inhibited by Cytosporone B, while silencing Nur77 can increase the protein expression level of phosphorylated IκB-α. After silencing IκB-α, both Cytosporone B and siNur77 did not affect pro-inflammatory genes and antioxidant stress. These findings reveal the first evidence that Nur77 exerts anti-inflammatory and antioxidant stress effects by inhibiting IκB-α phosphorylation expression in a Parkinson cell model. Nur77 may be a potential therapeutic target for Parkinson’s disease.

## INTRODUCTION

Parkinson’s disease (PD) is the second most common neurodegenerative disorder after Alzheimer’s disease (AD). PD is mainly characterized by dopaminergic system abnormalities in the substantia nigra. Although the underlying cause of dopaminergic neurodegeneration remains unclear, neuroinflammatory responses contribute to the pathogenesis of PD. Evidence suggests that inflammatory-related mediators, such as glutamate [1], interleukin-6 (IL-6) and tumor necrosis factor-α (TNF-α) [2], are critical to the pathogenesis of PD [3]. Moreover, increasing evidence suggests that Nur77 also plays a very important role in PD [4].

Nur77 is a member of the NR4A subfamily of nuclear receptors and plays a very important role in many biological processes. A serious of studies had shown that Nur77 was involved in the regulation of apoptosis [5], vascular disease [6], inflammation [7] metabolism [8, 9] and against neurodegeneration [10, 11]. Nur77 can reduce inflammatory responses through direct interactions with the p65 component of nuclear factor κB(NF-κB) [12]. Nur77 knockout mice significantly enhanced inflammatory responses characterized by a marked increase in IκB-α(NF-κB inhibitory protein) phosphorylation [13], which corresponds to elevated NF-κB activity. In contrast, Nur77 over expression decreases NF-κB activity. In atherosclerosis, Nur77 plays an anti-inflammatory role by activating macrophages [14]. Moreover, Nur77 expression has been detected in peripheral eosinophils from patients with atopic dermatitis [15].

Limited information is available regarding the role of Nur77 in PD. The recent studies demonstrated that Nur77 was involved in neurodegeneration [16, 17]. Abnormalities of Nur77 may induce dopaminergic imbalances in the brain [18]. Our study aimed to clarify both the role and mechanism of Nur77 in MPP^+^-treated PC12 cells and primary dopaminergic neurons.

## RESULTS

### Nur77 decreased intracellular LDH release and enhanced cell viability in MPP+-treated PC12 cells

**Figure 1:**
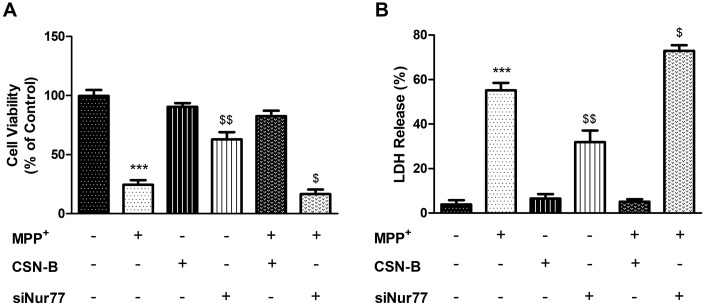
**Effects of Nur77 on PC12 cell viability and LDH.** (**A**) Nur77 increase PC12 cell survival and (**B**) decrease level of Lactate dehydrogenase (LDH) (^***^P < 0.001 compared with control group; ^$$^*P* < 0.01, ^$^ *P* < 0.05 compared with MMP^+^ group; n=3, mean +/- SEM).

To investigate the effects of MPP^+^ and Nur77 on PC12 cells, we exposed cells to MPP^+^(1.0 mM) or CSN-B (10mg/ml) for 24 h and/or siNur77 and examined cell survival using a MTT assay. In the MPP^+^-treated group, the MTT value in Figure 1A decreased significantly from 99.70±4.933% to 24.43±3.368% compared with the controls. In contrast, CSN-B mitigated this reduction and yielded a value of 62.83±6.129%. Interestingly, transfection with siNur77 led to a significant decrease in the mean MTT value of 3.173±0.645%, compared to 24.43±3.368% with MPP^+^ alone. This result suggests that Nur77 exerts a significant protective effect on MPP^+^-treated PC12 cells. Moreover, the inhibition of Nur77 induced PC12 cell death under MPP^+^ stress.

LDH is released from cells after membrane disruption and is therefore used as an indicator of late cell death. In this experiment, increased LDH release in Figure 1B was observed in cultures of MPP^+^-treated PC12 cells (55.17±3.347%) relative to controls (3.87±1.972%), while treatment with CSN-B mitigated this MPP^+^-induced elevation (31.90±5.179%). This finding suggests a significant neuroprotective effect of CSN-B in our *in vitro* PD model. To confirm that the neuroprotection mediated by CSN-B in this model was associated with Nur77, we treated cells with siNur77 and observed increased LDH release (72.92±2.493%). Accordingly, the observed inhibitory effect of CSN-B appeared to be associated with Nur77.

### Nur77 decreased the expression of proinflammatory gene in MPP^+^-lesioned PC12 cells

**Figure 2:**
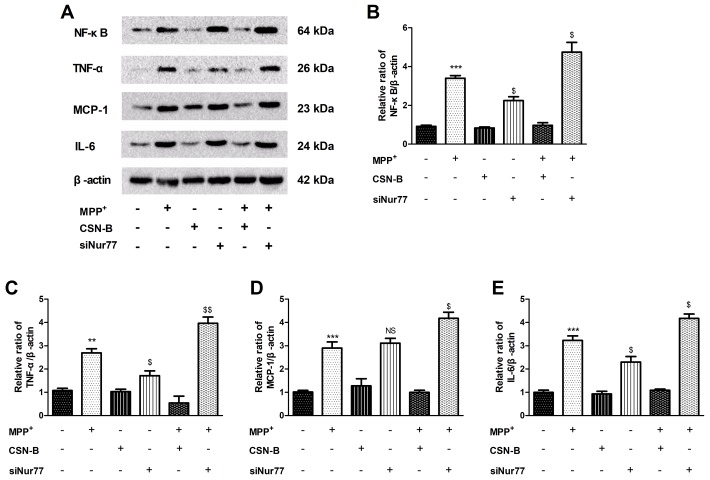
**Effects of Nur77 on cytokines expression in MPP^+^-treated PC12 cells.** (**A**) Nur77 increase NF-κB, TNF-α, IL-6 and MCP-1. (**B**–**E**) CSN-B and siNur77 treatment alone did not effect expression of NF-κB, TNF-α, IL-6 and MCP-1. MPP^+^ incubation pronouncedly increased level of NF-κB, TNF-α, IL-6 and MCP-1, while this elevation was abolished by CSN-B. (^***^P < 0.001,^**^P < 0.01 compared with control group; ^$$^*P* < 0.01, ^$^P < 0.05, NS = not significant. compared with MMP^+^ group; n=3, mean +/- SEM).

The pathogenesis of PD is accompanied by the strong expression or activation of various inflammatory factors Figure 2A, including NF-κB, TNF-α, IL-6 and MCP-1 Figure 2B–2E. Changes of inflammatory factors can affect neuronal activity and stress responses in surrounding tissues [19]. We used the classical Nur77 agonist CSN-B to induce strong expression of Nur77 prior to inflammatory factor production. We similarly observed the expression of inflammatory factors after treatment with siNur77. CSN-B and siNur77 treatment alone cann’t change expression of NF-κB, TNF-α, IL-6 and MCP-1. MPP^+^ incubation pronouncedly increased level of NF-κB, TNF-α, IL-6 and MCP-1 compared with controls, while this elevation was significantly abolished following CSN-B treatment. (Supplementary Figure 1). Silencing of Nur77 with siRNA significantly enhanced the expression of NF-κB, TNF-α, IL-6 and MCP-1 in MPP^+^-treated PC12 cells.

### Nur77 activates the Nrf2 signaling pathway and increase protein expression of HO-1 and NQO-1 in MPP+-lesioned PC12 cells

In PC12 cells, MPP^+^ treatment was accompanied by mitochondrial dysfunction and oxidative stress. These phenomena inhibited oxidative phosphorylation by inhibiting mitochondrial complex I, thus inducing changes in related oxidative stress indicators. Changes such as alterations in the Keap1/Nrf2 signaling pathway could decrease anti-oxidative stress damage in our PD model and alter corresponding factors, such as HO-1 and NQO-1. Thereby, neuronal survival or apoptosis is regulated by changes in anti-oxidative stress.

**Figure 3:**
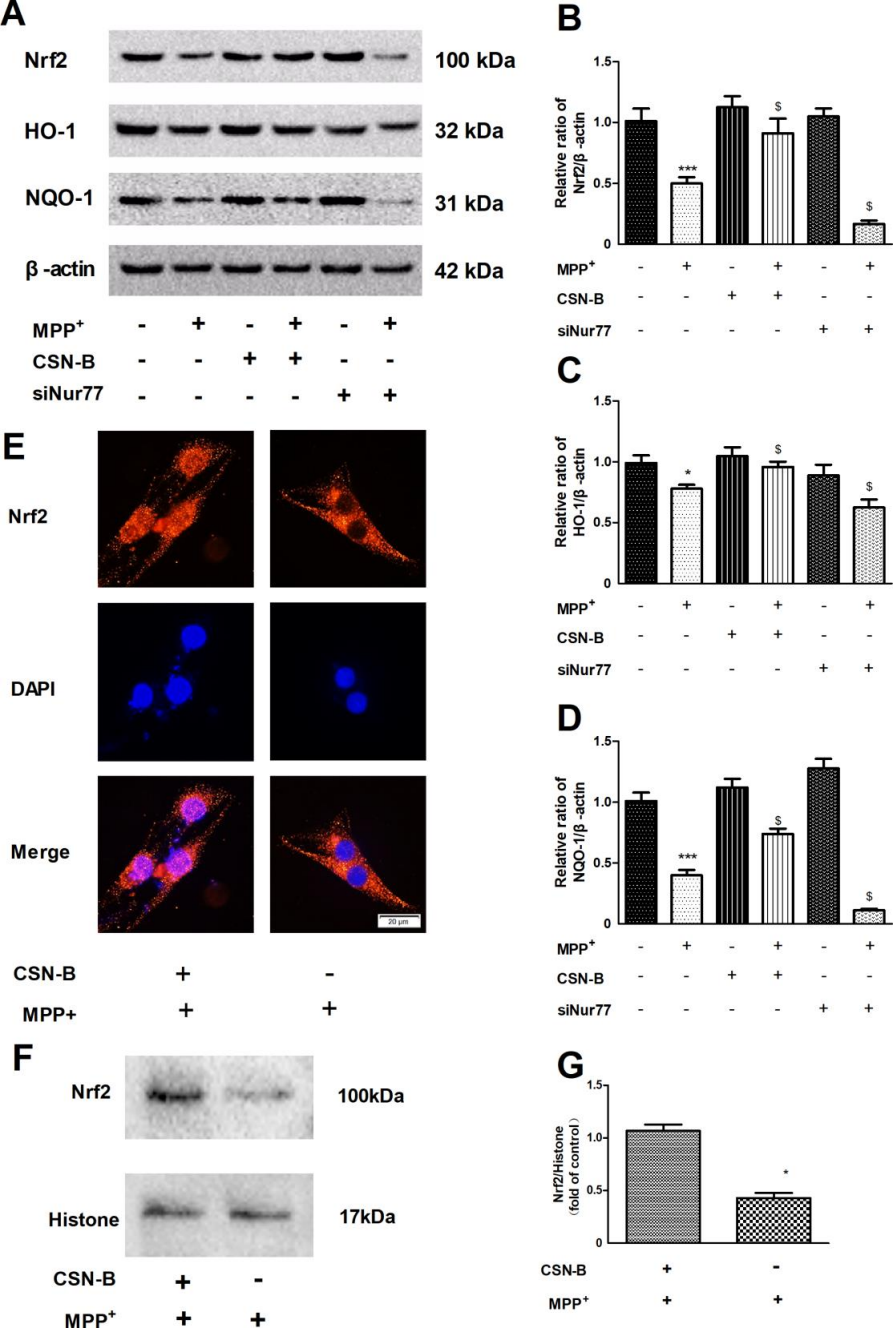
**Regulation of the Nur77 on anti-oxidant stress.** (**A**) CSN-B treatment significantly reversed the reduction of Nrf2 caused by MPP^+^, siNur77 transfection reduced the expression of Nrf2 under MPP^+^ stress. (**B**–**D**) CSN-B could significantly increase the expression of HO-1 and NQO-1, while Silencing of Nur77 with siRNA can significantly decrease HO-1 and NQO-1expression.(^***^P < 0.001, ^*^P < 0.05 compared with control group; ^$^*P* < 0.05.compared with MMP^+^ group; n=3, mean +/- SEM). (**E**) Immunohistochemistry of Nrf2 in PC12 cells after CSN-B treatment. (**F**, **G**) Protein expression and Quantitative analysis of Nrf2 in PC12 cell nucleus.

Our findings demonstrated a significant reduction in Nrf2 expression under MPP^+^ stress conditions when compared to the control level. However, CSN-B treatment significantly reversed the reduction of Nrf2 caused by MPP^+^, B. Furthermore, siNur77 transfection significantly reduced the expression of Nrf2 under MPP^+^ stress (Figure 3A, 3B). In response to CSN-B, translocation of Nrf2 from the nucleus to the cytoplasm was clearly observed (Figure 3E, 3F).

The anti-oxidative stress index HO-1 and NQO-1 is downstream of Nrf2. In MPP^+^-treated PC12 cells, CSN-B could significantly increase the expression of HO-1 and NQO-1, while Silencing of Nur77 with siRNA can significantly decrease HO-1 and NQO-1expression in Figure 3A, 3C, 3D. After silencing of Nur77, CSN-B couldn’t significantly increase the protein expression of Nrf2, HO-1 and NQO-1 (Supplementary Figure 2). We examined the nuclear-plasma transfer of Nrf2 and found that CSN-B could promote the activated Nrf2 in nuclear, which was also supported by the protein quantitative analysis of Nrf2 nuclear protein in Figure 3E–G.

### Inhibition of NF-κB can decrease the TNF-α, MCP-1 and IL-6 expression induced by MPP^+^

**Figure 4:**
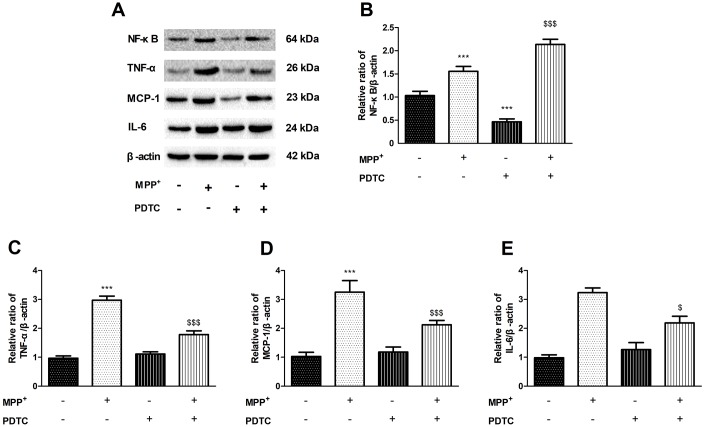
**The effect of of PDTC on cytokines expression.** (**A**–**E**) NF-κB specific inhibitor PDTC can decrease the proteins expression of NF-κB, TNF-α, MCP-1 and IL-6 in MPP^+^-treated PC12 cells. (^***^P < 0.001, compared with control group; ^$$$^*P* < 0.001.compared with MMP^+^ group; n=3, mean +/- SEM).

To confirm inflammatory response is caused by NF-κB signaling pathway, we used the NF-κB specific inhibitor PDTC to investigate changes of the inflammatory factor (Figure 4A). The expression of TNF-α, MCP-1 and IL-6 significantly increased in MPP^+^-treated PC12 cells compared to control group, while this elevation was significantly inhibited following PDTC treatment (Figure 4B–4E).

### Nur77 inhibited the expression of NF-κB by decreasing the phosphorylation of IκB-α in MPP^+^-treated PC12 cells

**Figure 5:**
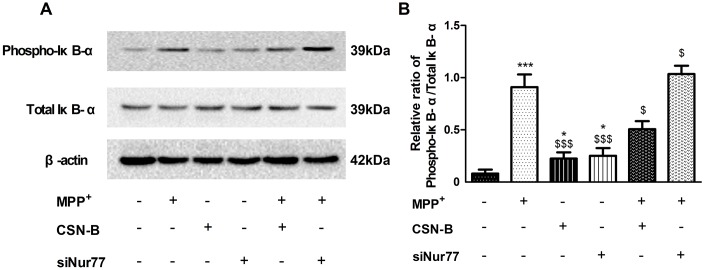
**The effect of cytosporone B and siNur77 on protein expression of phosphorylation of IκB-α.** (**A**, **B**) MPP^+^ and siNur77 can increase the level of the phosphorylation IκB-α in MPP^+^-treated PC12 cells, while CSN-B reverse this increase. (^***^P < 0.001 compared with control group; ^$$$^*P* < 0.001, ^$^*P* < 0.05, compared with MMP^+^ group; n=3, mean +/- SEM).

In recent studies of neurodegenerative diseases, especially AD and PD [21], the regulation of NF-κB is considered a key pathological element. Our study found that phosphorylation IκB-α was significantly increased in MPP^+^-treated PC12 cells, while CSN-B mitigated this increase. Moreover, we also observed significantly elevated levels of phosphorylated IκB-α in siNur77-treated PC12 cells relative to the control group in Figure 5A, 5B.

### Nur77 can inhibit inflammatory responses and oxidative stress in primary neurons

**Figure 6:**
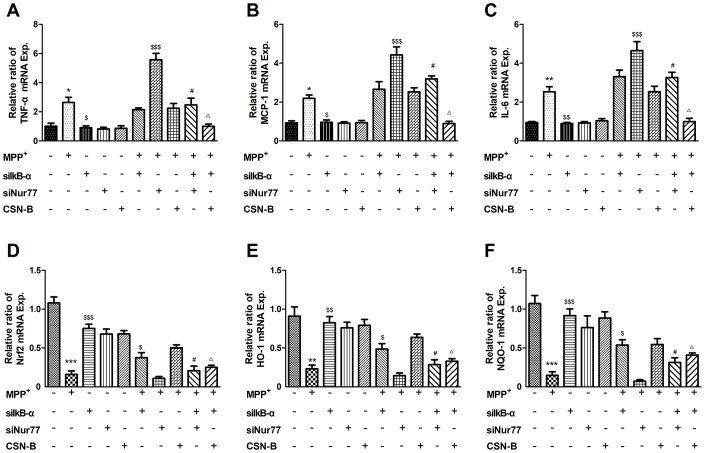
**Effects of Nur77 on cytokines and oxidant stress in primary neurons.** MPP^+^ significant upregulated in the expression of mRNAs encoding (**A**–**C**) TNF-α, IL-6 and MCP-1 and downregulated (**D**–**F**) Nrf2, HO-1, NQO-1, while siIκB-α can reverse the upregulation and downregulation. The role of CSN-B is the same as siIκB-α, while siNur77 can significantly increase level of TNF-α, IL-6 and MCP-1 and decrease level of Nrf2, HO-1, NQO-1. When IκB-α is silenced by IκB-α siRNA, the regulative role of CSN-B and siNur77 is significantly reduced (^***^P < 0.001; compared with control group; ^$$$^*P* < 0.001,^$$^ *P* < 0.01,^$^*P* < 0.05, compared with MMP^+^ group; ^#^*P*<0.05 compared with SiNur77+MPP^+^ group; ^Δ^*P*<0.05 compared with CSN-B+MPP^+^ group; n=3, mean +/- SEM).

To further demonstrate that Nur77 attenuates inflammatory responses and oxidative stress by phospho-IκB-α, primary neurons were treated with MPP^+^, the Nur77 agonist cytosporone B, and/or small interfering RNAs (siRNAs) against Nur77 and siIκB-α. We found that MPP^+^ significant increased the expression of mRNAs encoding TNF-α, IL-6 and MCP-1 and decreased Nrf2, HO-1, NQO-1, while siIκB-α can reverse the changes. The role of CSN-B is the same as siIκB-α, while siNur77 can significantly increase level of TNF-α, IL-6 and MCP-1 and decrease level of Nrf2, HO-1, NQO-1. These results prove that Nur77 can inhibit inflammatory responses and oxidative stress in MPP^+^ treated primary neurons (Figure 6).

## DISCUSSION

In our study, MPP^+^-treated significantly increased the level of TNF-α, MCP-1 and IL-6 of PC12 cells, but CSN-B could reduce the expressive level of TNF-α, MCP-1 and IL-6, which demonstrated that CSN-B can inhibit inflammatory response. The model of MPP^+^ stress-induced PD is often associated with oxidative stress damage because MPP^+^ inhibits mitochondrial complex I and thus interferes directly with cellular oxidative phosphorylation. This damage represents a common type of pathological injury during the course of PD [20]. Therefore, PD research must also address the repair of oxidative stress damage. MPP^+^-induced oxidative stress is affected by the anti-oxidative stress response induced by Nrf2/Keap1/ARE pathway activation [21]. Our study also showed that CSN-B can upregulate Nrf2, HO-1, NQO-1 and exert anti-oxidative stress response, while silencing of Nur77 with siRNA increased the protein expression levels of TNF-α, MCP-1, IL-6 and decreased Nrf2, HO-1, NQO-1 in MPP^+^-lesioned PC12 cells. Cytosporone B can target the ligand binding domain of Nur77, which selectively stimulates the transactivational activity of Nur77, which strongly suggests that Nur77 can inhibit inflammatory response and oxidative stress in MPP^+^-treated PC12 cells.

Interestingly, we also found that CSNB and SiNur77 alone had no effect on NF-κB (P65), but in MPP^+^ treated PC12 cells, CSNB inhibited MPP^+^-induced upregulation of NF-κB (P65), and SiNur77 increased NF-κB (P65) expression compared with MPP^+^ group, which was consistent with the expression trend of inflammatory factors. So we speculated if the anti-inflammatory properties of Nur77 were attributed to the inhibition of NF-κB signal pathway in neurons. To further prove that the inflammtory response caused MPP^+^ is contribute to NF-κB pathway activity, we inhibited the expression of NF-κB using PDTC (NF-κB inhibitor) in MPP^+^-treated PC12 cells and observed a significant downregulation of TNF-α, MCP-1and IL-6.

NF-kB play a critical role in inflammatory responses. In resting cells, NF-kB is sequestered in the cytoplasm by the IκB family, including IκB-a and IκB-b. IκB-α protein can be activated via pro-inflammatory cytokines. This process leads to the phosphorylation of IκB via the IκB kinase complex [22]. Once phosphorylated IκB protein is ubiquitinated and rapidly degraded by the proteasome, which releases NF-κB from IκB. NF-κB then translocates to the nucleus to initiate transcription by binding to many specific gene promoter elements [23, 24]. Here, we measured the phosphorylation of IκB-α and found that phosphorylation of IκB-α significantly increased, CSN-B can decrease level of phosphorylation of IκB-α and silencing of Nur77 with siRNA can increased level of phosphorylation of IκB-α, which also demonstrated that Nur77 can decrease the phosphorylation of IκB-α in MPP^+^-lesioned PC12 cells. The decrease of IκB-α phosphorylation lead to sustained binding of this inhibitory molecule to NF-κB, which would block NF-κB nuclear translocation and activity. In primary neurons, we also found that Nur77 can decrease mRNA level of TNF-α, MCP-1, IL-6, and increase mRNA level of Nrf2, HO-1, NQO-1, and the regulation of Nur77 on inflammatory response and oxidative stress was significantly alleviated after silencing IκB-α of with siRNA.

In summary, these findings revealed that Nur77 exerts a neuroprotective effect against dopaminergic neurodegeneration by inhibiting phospho-IκB-α mediated anti-inflammatory and anti-oxidative stress mechanisms. To our best knowledge, this study is the first to demonstrate that Nur77 regulates inflammatory response and oxidative stress by inhibiting the phosphorylation of IκB-α in PD cell model. These results help to clarify relationships among Nur77, neuroinflammation and dopaminergic system, and provide a new prospect on PD.

## CONCLUSIONS

Our results provide the evidence that Nur77 attenuates MPP^+^-mediated inflammatory responses and oxidative stress by inhibiting phosphorylation of IκB-α in PD cell model. These findings enhance our understanding of the critical role of Nur77 in the treatment of neurodegenerative disorders, such as PD.

## MATERIALS AND METHODS

### Cell culture and treatments

PC12 rat adrenal pheochromocytoma cells were maintained routinely in RPMI-1640 medium supplemented with 5% fetal bovine serum, 10% horse serum, 100 U/ml benzyl penicillin and 100 mg/L streptomycin (Gibco, USA). The cells were cultured at 37ºC in a humidified atmosphere containing 5% CO_2_. The cells were fed every 2–3 days and sub-cultured once they reached 80–90% confluence. Primary neurons were conducted as previously described [25]. Newborn C57BL/6J mice were obtained within 24 h of birth from HFK Bioscience Co., Ltd. (Beijing, China). Isolated neurons were cultured in DMEM containing F-12 and 1 mM glutamine. The primary culture neurons were maintained at 37°C in an incubator containing 5% carbon dioxide.

The cells were treated with MPP^+^ (Sigma-Aldrich, **≥97%,** M7068-10MG,500μM), the Nur77 agonist cytosporone B (CSN-B, Santa Cruz Biotechnology, ≥98%, sc-252653, 10mg/ml), PDTC (Santa Cruz Biotechnology, ≥98%, sc-203224, 300 mM) and/or small interfering RNAs (siRNAs) against Nur77 (siNur77, Santa Cruz Biotechnology, sc-36109) or IκB-α (siIκB-α, Santa Cruz Biotechnology, sc-29361). For all of the experiments, the cells were seeded in 96-well or 6-well plates at a density of 1.0×10^5^ cells/ml and incubated for 24 h.

### MTT cell viability assay

PC12 cell viability after MPP^+^, Cytosporone B(CSN-B) and Nur77 siRNA treatment was evaluated using the MTT assay. Briefly, PC12 cells were grown in 96-well plates (100 μl/well) at a density of 1×10^5^ cells/ml. After incubation, the cells were washed once with phosphate-buffered saline (PBS), after which100 μl of serum-free medium containing MTT (final concentration, 0.5 mg/ml) were added to each well. After a 4-h incubation, the supernatant was removed from each well, the cells were washed twice with PBS and the formazan product was dissolved in 100 μl of dimethylsulfoxide (DMSO). The absorbance in each well at a wavelength of 570 nm was read using a microplate reader. The results for each experimental condition are expressed as percentages of the control group results.

### LDH assay

Cell viability was also measured by determining the release of LDH into the culture medium following damage to the cellular membranes. Total LDH activity was measured using the Lactate Dehydrogenase Activity Assay Kit (Sigma-Aldrich, MAK066-1KT, USA) according to the manufacturer’s instructions. The absorbance in each culture well at 490 nm was measured using an automatic microplate reader. The data are presented as percentages of LDH in the MPP^+^ group, which was set at 100%.

### siRNA silencing

PC12 cells and primary neurons were transfected with siNur77 and siIκB-α in 0.5μg of Lipofectamine 3000 according to the manufacturer’s instructions. For each transfection in a 6-well plate, 2–8 μl of the siRNA duplex (0.25–1 μg or 20–80 pmol siRNA) were diluted in each well. (Supplementary Figure 3)

### Immunocytochemistry

PC12 cells were seeded on circular slides with suitable cell processing in a suitable well plate and fixed with paraformaldehyde. The cells were then labeled with a primary antibody specific to Nrf2 (1:200 dilution). After a subsequent incubation with an Alexa Fluor 555-conjugated secondary antibody (1:500 dilution), the labeled cells were observed using an LSM 780 confocal microscope (Zeiss, Germany).

### Quantitative real-time RT-PCR

For quantitative real-time RT-PCR, total RNA was extracted from the cells using TRIzol (Invitrogen, USA) according to the manufacturer’s protocol. Subsequently, cDNA was synthesized using the Advantage RT for PCR kit (BD Biosciences, USA) and oligo (dT) primers. Next, target genes were PCR amplified and quantified using the SYBR Green PCR Master Mix (Applied Biosystems, USA), and the results were normalized to the expression of GAPDH mRNA. All experiments were performed in triplicate and repeated at least three times.

### Western blotting analysis

After pretreatment with CSN-B and/or MPP^+^ and/or siNur77, the cells were rinsed thrice with ice-cold PBS and incubated in ice-cold lysis buffer for 20 min on ice. The supernatants were then collected and subjected to a bicinchoninic acid assay to determine the protein concentrations. The samples were then mixed with 5× Loading buffer and heated for 5 min at 100ºC. Next, aliquots with equivalent amounts of total protein (30 μg) were separated on NuPageBis-Tris 10% gels (Invitrogen, USA) and transferred to polyvinylidene fluoride (PVDF) membranes. The PVDF membranes were incubated overnight at 4ºC in solutions with primary antibodies (1:2000) against TNF-α, IL-6, MCP-1(Abcam, Cambridge, UK), and (1:2000) against Nrf2, HO-1, NQO-1, NF-κB p65 and Phospho IκB-α (Cell Signaling Technology, Danvers, MA), and primary antibodies (1:5000) against β-Actin (CWBio, Beijing, China), washed thrice and incubated with horse radish peroxidase-conjugated secondary antibodies. Negative controls were prepared by excluding the primary antibodies. Images of the labeled membranes were analyzed using Image J software (National Institutes of Health, USA).

### Statistical analysis

The data are expressed as means ± standard errors of the means. The results of MTT and LDH assays and Western blot quantifications of different proteins were analyzed using a one-way analysis of variance, followed by Tukey’s post hoc analysis (SPSS 15.0; IBM, USA). *P* values of < 0.05 were regarded as statistically significant.

## Supplementary Material

Supplementary Figures
