# Supplementary material for: Nur77 attenuates inflammatory responses and oxidative stress by inhibiting phosphorylated IκB-α in Parkinson’s disease cell model
Source: Aging (Albany NY). 2020 May 13;12(9):8107–19. doi: 10.18632/aging.103128 (PMC7244064; doi:10.18632/aging.103128)
Supplement: Supplementary Figures [file aging-12-103128-s001..pdf]

SUPPLEMENTARY FIGURES

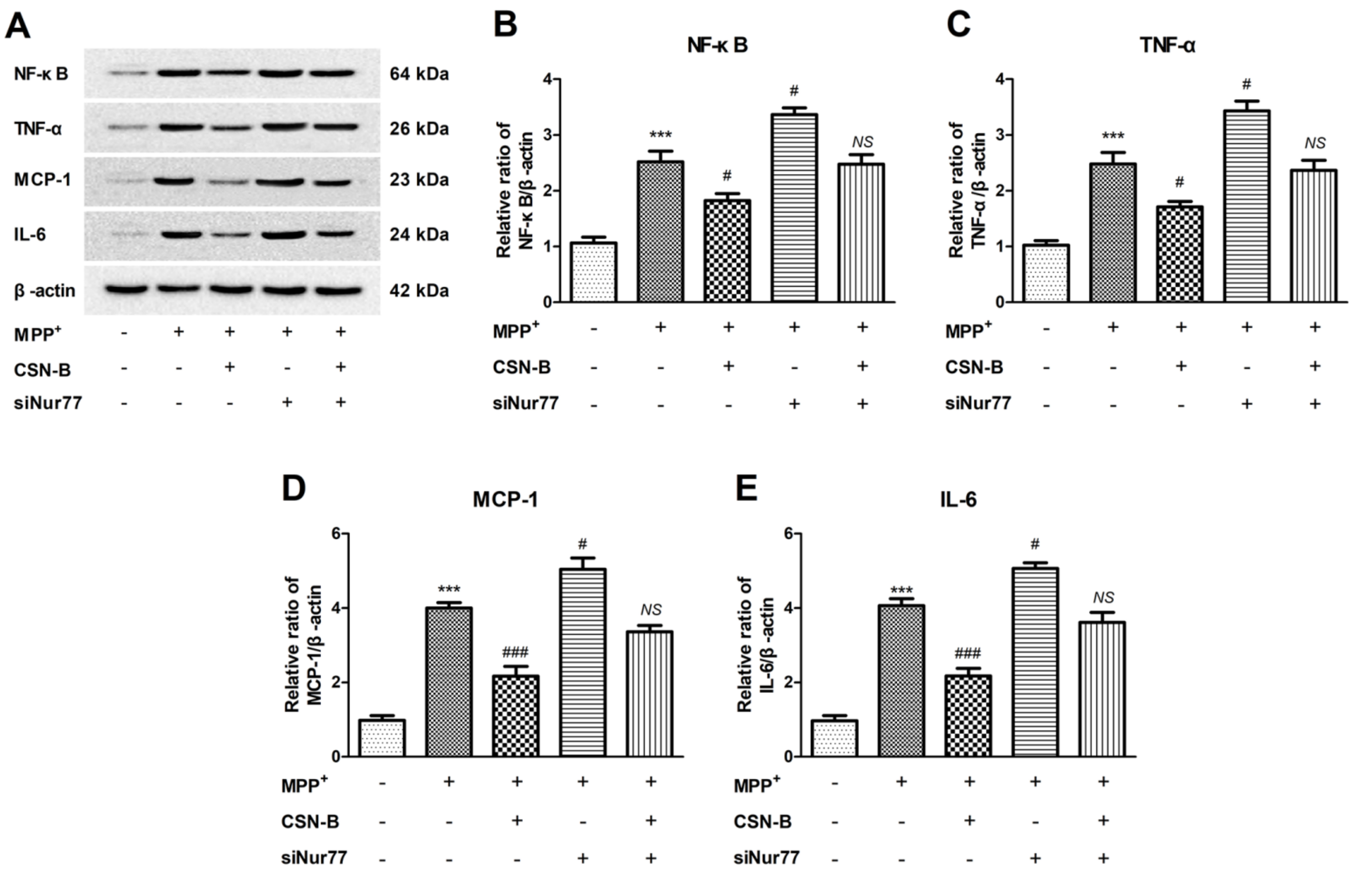

Supplementary Figure 1. CSN-B decrease proinflammatory genes by increasing Nur77 in MPP+-treated PC12 cells. MPP^+ incubation pronouncedly increased level of NF-κB, TNF-α, IL-6 and MCP-1, while this elevation was abolished by CSN-B, the treatment of siNur77 increased this effect. (**A**) Effects of NF-κB, TNF-α, MCP-1, IL-6 on cytokines expression in MPP^+, CSN-B and siNur77 treated PC12 cells. (**B**–**E**) Quantitative analysis of protein expression of NF-κB, TNF-α, MCP-1, IL-6. Results are expressed as the relative expression of β-actin (****P*<0.001 compared with control group; $^{###}$*P*<0.001,$^{\#}$*P*<0.05, compared with MMP^+ group; *NS* means not significicant; n=3, mean +/- SEM).

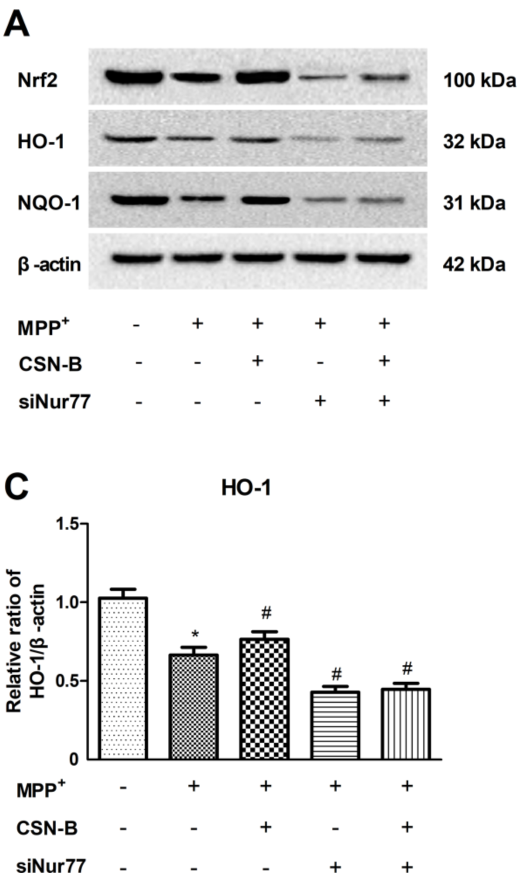

Supplementary Figure 2. CSN-B enhance oxidant stress by increasing Nur77 in MPP+-treated PC12 cells. CSN-B treatment significantly increase the level of Nrf2,HO-1 and NQO-1compared to MPP^+ group, Silencing of Nur77 with siRNA significantly reduced the expression of Nrf2, HO-1 and NQO-1 under MPP^+ stress. After Silencing of Nur77 with siRNA, CSN-B incubation couldn't significantly increase the protein expression of Nrf2, HO-1 and NQO-1. (**A**) Effects of Nrf2, HO-1, NQO-1 on cytokines expression in MPP^+, CSN-B and siNur77 treated PC12 cells. (**B**–**D**) Quantitative analysis of protein expression of Nrf2, HO-1, NQO-1. Results are expressed as the relative expression of β-actin. (***P*<0.01, **P*<0.05 compared with control group; $^{\#}$*P*<0.05compared with MMP^+ group; n=3, mean+/- SEM).

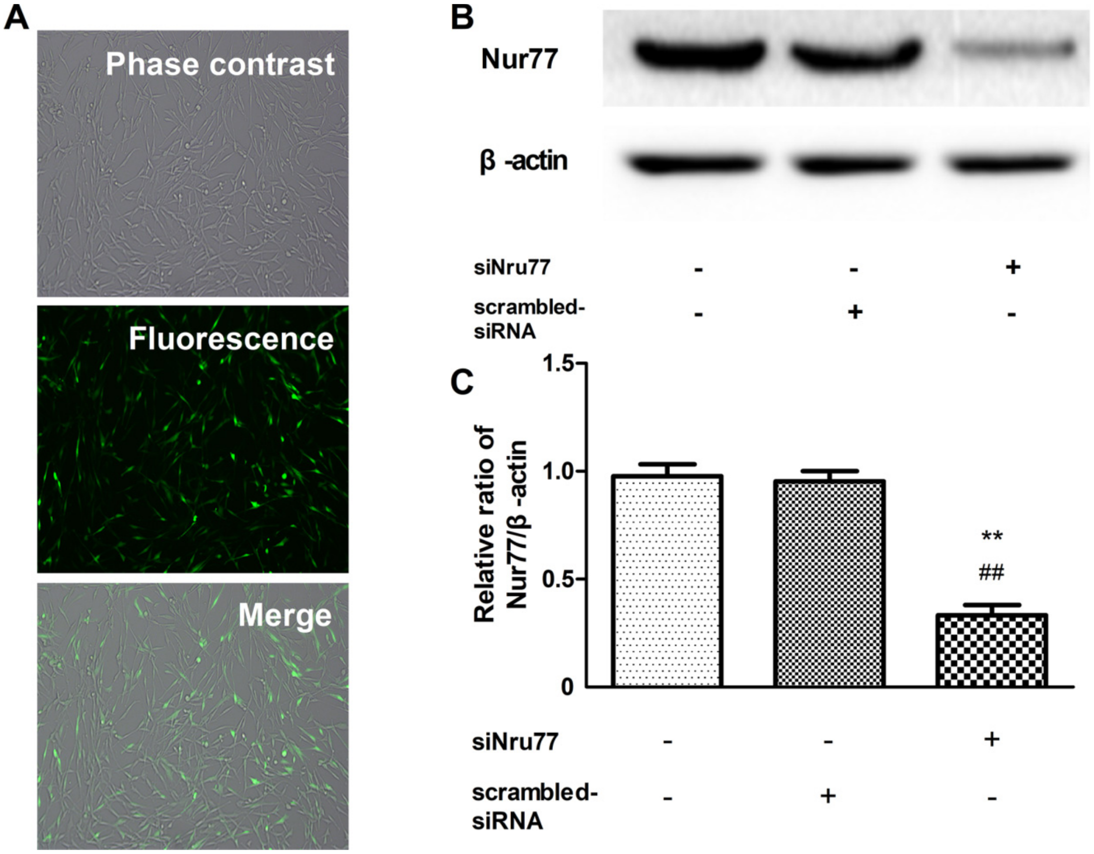

Supplementary Figure 3. Silencing efficiency detection of siNur77. (**A**) Silence effect of siNur77 in PC12 cells. Phase contrast shown all PC12 cells, fluorescence indicate PC12 cells after silencing Nur77. (**B**) Effects of Nur77 on cytokines expression in siRNA treated PC12 cells. (**C**) Quantitative analysis of protein expression of Nur77. Results are expressed as the relative expression of β-actin. Data are expressed as mean ± SEM; $^{**}p$< 0.01versus control group, $^{\#\#}p$< 0 .01versus scrambled siRNA treatment group.
